# A follow-up study of early intensive behavioral intervention program for children with Autism in Syria

**DOI:** 10.1038/s41598-022-27198-4

**Published:** 2023-01-02

**Authors:** Wissam Mounzer, Donald M. Stenhoff, Jamal M. Alkhateeb, Amal J. Al Khatib

**Affiliations:** 1 Department of Psychology, Damascus University, Damascus, Syria; 2 Department of Psychology, Arizona State University, Tempe, AZ USA; 3 Department of Special Education, The University of Jordan, Amman, Jordan; 4 Department of Early Childhood Education, Queen Rania Faculty for Childhood, Hashemite University, Zarqa, Jordan; 5 Present Address: Department of Special Education, Stockholm University, Stockholm, Sweden

**Keywords:** Human behaviour, Evolution

## Abstract

We examined the sustained effects of early intensive behavioral intervention (EIBI) on 66 children with autism spectrum disorder who participated in the Future Center’s EIBI program. Children were assessed using the childhood autism rating scale (CARS), autism behavior checklist (ABC), and adaptive behavior scale (ABS-Arabic) 3 years after leaving the program. Continued positive effects were observed in several areas, including adaptive behavior and autism symptoms. However, participants’ social skills might have declined on the ABS-Arabic after service withdrawal. Additionally, a significant negative association (*p* < .001) was found between participants’ performance on the CARS and the number of weekly trials. This is encouraging, given the lack of EIBI services and regional instability. Future research should increase the sample size and use a more rigorous design.

## Introduction

Children with autism spectrum disorder (ASD) show dramatic improvement when provided with early intensive behavioral interventions (EIBI) grounded in applied behavior analysis (ABA; Howlin^1^). Several researchers have reported the effectiveness of EIBI (e.g., Ben-Itzchak & Zachor^2^; Eikeseth et al*.*^3^; Eldevik et al*.*^4^; Estes et al*.*^5^; Lovaas^6^; Peters-Scheffer et al*.*^7^; Reichow et al*.*^8^; Sheinkopf & Siegel^9^; Warren et al*.*^10^), whereas others found that EIBI has a “moderate” effect on adaptive behavior in children with ASD^11–17^. The degree to which these outcomes are sustained over time is largely unknown, and further research is required to include larger and more representative demographic samples^18–20^. In a review of 11 studies published from 1987 to 2007, Howlin et al*.*^21^ reported mixed results regarding the effects of EIBI in children with ASD and that any improvements are likely to be greatest in the first year of intervention.

Longitudinal research investigating the lives of adults who received ABA-based therapy since childhood is limited^22–25^. However, Steinhausen et al*.*^26^ indicated that while the short-term effects of intervention programs, such as Treatment and Education of Autistic and related Communications Handicapped Children (TEACCH) and ABA-based interventions, have been well evaluated, their long-term effects remain unknown. Steinhausen et al. reviewed a total of 22 studies; of these, only three performed a true follow-up as opposed to only a post-test. The shortest post-test was 6 months, whereas the longest was a true follow-up study of up to 9 years^27^. Estes et al*.*^5^ reported evidence that gains from EIBI are maintained 2 years later in overall intellectual ability, adaptive behavior, and symptom severity. Smith et al*.*^51^ reported that EIBI gains were maintained at follow-up, 10 years after the EIBI had ended. Participants also showed a significant reduction in autism symptoms between intake and follow-up. While some systematic reviews and meta-analyses found limited evidence on the significant long-term effect of EIBI (Howlin et al.^21^; Reichow et al.^8^), many other reviews reported long-term benefits of EIBI for children with ASD and recommend EIBI as an intervention of choice for these children (Daniolou et al.^62^; Eldevik et al.^4^; Frazier et al.^63^; Smith et al.^51^). However, the^64^ found that 76% of TRICARE beneficiaries in the Autism Care Demonstration show little to no change in symptom presentation over the course of 12 months of ABA services, with an additional 9% demonstrating worsening symptoms. However, the report concurs that ABA research has not established a dose–response relationship between severity, treatment needs, and intensity of services. Additionally, ABA services may be one component of a comprehensive treatment plan for a beneficiary diagnosed with ASD. These findings raise a crucial question regarding whether ABA-based services will continue to have positive effects on children in several areas, including adaptive behavior and autism symptoms.

Practitioners and researchers must also consider that behavioral research is largely based on data from Western, educated, industrialized, and democratic countries. Thus, research that has been interpreted as universal may be specific to that culture^28^. The field of ABA has emphasized the importance of working for social justice and for developing sensitivity and humility toward other cultures and cultural expressions^29^. However, these aspects have not always been considered in practice^30^. Miller et al*.*^30^ described how a changing world, with increasing diversification of ethnicities and social gaps, places new demands on behavioral analysts regarding cultural sensitivity.

### ABA-based interventions for Syrian children with ASD

ABA-based services are still in their infancy in the Middle East, and limited research has been conducted regarding these interventions (e.g., Al-Hemoud & Al-Asfoor^31^; Eapen et al*.*^32^; Hussein & Taha^33^; Kelly et al*.*^34^; Sartawi^35^). In Syria, organizational efforts are necessary to increase the application of ABA services^66^. In October 2003, an Association for Behavior Analysis International (ABAI) delegation reported a need for ABA services and higher education opportunities in the Gulf Cooperation Council in the Middle East^36^. To date, information on the use of EIBI, the standards of implementation of ABA programs, and the types of services offered in Syria are lacking. The credentials, clinical training, and quality of services vary greatly from clinic to clinic; ABA-based interventions are often implemented in conjunction with other programs in an eclectic model^65^. A 2013 review of ABA programs in several settings revealed that ABA programs were not monitored by an onsite board-certified behavior analyst. However, no follow-up reports were found on whether this situation is still observable today. To further exacerbate these issues, Syria has been engaged in a civil war since 2011, limiting access to information regarding ASD and ASD-related services^65^.

Despite the knowledge about EIBI programs for children with ASD in American and European nations, there are no longitudinal studies regarding EIBI interventions in young children with ASD in Syria. Syria is still in a state of civil unrest, and the situation remains volatile. However, reconstruction attempts have been recently initiated in the country, suggesting that the security situation in the country will improve shortly. However, many Syrian children have been affected by brutality, displacement, loss of or separation from family members, and lack of access to vital services (United Nations International Children’s Emergency Fund [UNICEF], 2019). Thus, despite the interventions being implemented to address children’s issues, the country still needs to develop plans of action that specifically target children with developmental disorders. In the current study, we aimed to assess the extent of the sustained effects of EIBI delivered by the Future Center (FC-EIBI), 3 years after the intervention was terminated, when the children were an average of 8 years old. The current study reports on a group of children who received, on average, 2 years of EIBI. The participants in this study were children who participated in a previously published study^45^. In 2013, many of our participants were displaced due to the civil war, which impacted the services that these children received, as well as adaptive behavior and severity of autism symptoms, according to their parents. In particular, we aimed to answer the following questions: (a) after the 3-year FC-EIBI program, were there changes in participants’ scores for the autism behavior checklist (ABC; Volkmar et al*.*^37^), childhood autism rating scale (CARS^52^; Schopler et al.^38^), and adaptive behavior scale-Arabic (ABS-Arabic^53^;? (b) Are there differences in place of residence regarding these program outcomes? (c) Is there a statistically significant relationship between opportunities to respond (i.e., trials) and participants’ follow-up scores for the ABS-Arabic and those for the ABC and CARS? The opportunities to respond may vary from session to session. Thus, we elected to use trials as it directly quantifies the treatment the participant received rather than hours and it correlates to learning (Fisher & Berliner^55^; Kestner et al.^54^; Ross & Greer^56^).

## Results

We initially compared the total scores for the CARS, ABC, and ABS-Arabic pre-, post-, and follow-up tests. On average, the children performed worse in the 2008 pretest, 2010 post-test, and directly after intervention than in the 2013 follow-up test, which was 3 years after the intervention. Repeated measures ANOVAs were conducted with time point as a within-subjects factor.

### CARS, ABC, and ABS-Arabic outcomes

There was a significant main effect of the time point of the FC-EIBI program on participants’ CARS, ABC, and ABS-Arabic scores, *F*_*CARS*_ (1.87, 121.31) = 442.46, *p* < .001 *η*^2^ = 0.87; *F*_*ABC*_ (1.60, 103.98) = 398.59, *p* < .001 *η*^2^ = 0.86; *F*_*ABS-Arabic*_ (1.62, 105.32) = 755.53, *p* < .001 *η*^2^ = 0.91(Table 1).

**Table 1:** Descriptive Statistics of Pre-. Post. and Follow-up Tests (n = 66).

	Time	Mean	SD	SE	95% CI		*F*	*P*	*η*^*2*^
					Lower bound	Upper bound			
CARS pretest	1	36.24	4.91	0.60	35.03	37.44	442.46	.000	0.871
CARS. posttest	2	32.82	5.684	0.70	31.42	34.21			
CARS follow-up	3	30.92	5.62	0.69	29.54	32.30			
ABC. pretest	1	118.68	29.11	3.58	111.52	125.84	398.59	.000	0.863
ABC. posttest	2	95.80	29.54	3.64	88.54	103.06			
ABC. follow-up	3	84.24	27.34	3.37	77.52	90.96			
ABS-Arabic pretest	1	74.74	49.02	6.03	62.69	86.79	755.53	.000	0.912
ABS-Arabic posttest	2	260.76	54.81	10.15	204.50	245.06			
ABS-Arabic follow-up	3	224.79	82.49	6.74	247.28	274.23			

*ABC* autism behavior checklist, *CARS* childhood autism rating scale, *ABS-Arabic* adaptive behavioral scale-Arabic.

*N*: total number of individuals in the sample.

*F*: variance of the group means.

*P*: probability that measures the evidence against the null hypothesis.

*η*^2^: effect size.

**Figure 1:**
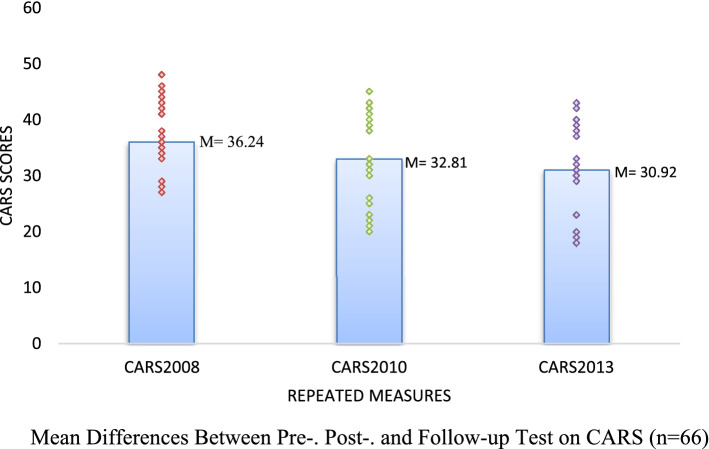
Mean Differences Between Pre-. Post-. and Follow-up Test on CARS (n = 66).

Bonferroni-adjusted pairwise comparisons showed that post-test scores of both CARS (*M* = 32.81, *SE* = 0.70), and ABC (*M* = 95.80, *SE* = 3.63) were lower than the scores of the pretest scores of CARS (*M* = 36.24, *SE* = 0.60), and ABC (*M* = 118.68, *SE* = 3.58). This improvement was statistically significant (*p* < .001). In addition, both CARS and ABC scores at three years follow-up (CARS; *M* = 30.92, *SE* = 0.69, ABC; *M* = 84.24, *SE* = 3.36) were significantly lower than scores before (*p* < .001) and after (*p* < .001) the FC-EIBI program. The results are shown in Supplementary Table 2 and Fig. 1.

**Figure 2:**
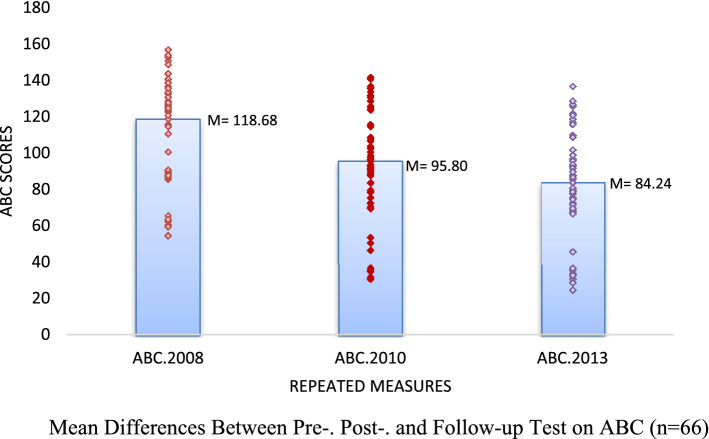
Mean Differences Between Pre-. Post-. and Follow-up Test on ABC (n = 66).

**Figure 3:**
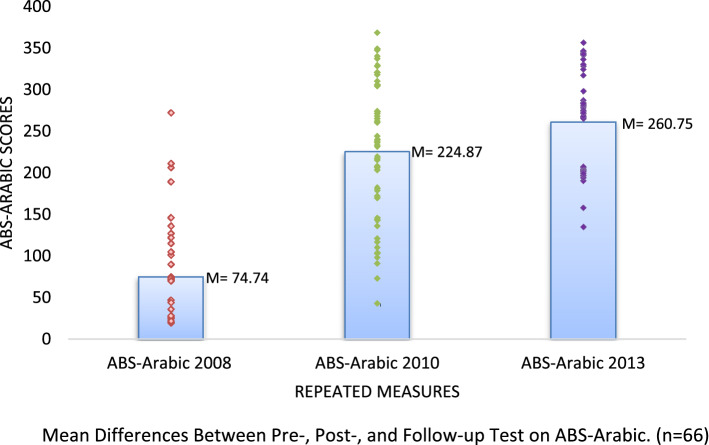
Mean Differences Between Pre-, Post-, and Follow-up Test on ABS. (n = 66).

Furthermore, the post-test scores of ABS-Arabic (*M* = 224.78, *SE* = 10.15) were higher than the pretest scores (*M* = 74.74, *SE* = 6.03). This improvement was statistically significant (*p* < .001). Additionally, ABS-Arabic scores at the 3-year follow-up (*M* = 260.75, *SE* = 6.74) were significantly lower than scores before (*p* < .001) and after (*p* < .001) the FC-EIBI program. The results are shown in Supplementary Table 2 and Fig. 3*.*

We analyzed the scores for the CARS, ABC, and ABS-Arabic by place of residence (i.e., forced displacement, original home; Supplementary Table 3) by using the independent samples t-test. On average, the displaced children performed worse (*M*_CARS_ = 33.76, *SD*_CARS_ = 5.57; *M*_ABC_ = 99.52, *SD*_ABC_ = 25.98; *M*_ABS_ = 288.19, *SD*_ABS_ = 43.92) than the children living in their original home (*M*_CARS_ = 28.70, *SD*_CARS_ = 4.62; *M*_ABC_ = 72.27, *SD*_ABC_ = 22.11; *M*_ABS_ = 225.7, *SD*_ABS_ = 47.22). Differences were statistically significant across all scales: CARS = 5.05, ABC = 27.24, ABS-Arabic = 62.43. *t*_CARS_ (65) = 4.02, *p* < .001; *t*_ABC_ (65) = 4.60, *p* < .001; *t*_ABS_ (65) = 5.54, *p* < .001).

We also used the Spearman's Rank correlation coefficient to assess the relationship between the number of weekly trials (no trials/week; < five trials/week; and more than five trials/week) and participants’ follow-up scores for the CARS, ABC, and ABS-Arabic (Supplementary Table 4). A strong significant negative correlation was found between the number of weekly trials and participants’ scores for the CARS and ABC: *r*_CARS_ = -0.78, *p* < .001, *N* = 66; *r*_ABC_ = -0.81, *p* < .001, *N* = 66. A strong significant positive correlation was also observed between the number of weekly trials and participants’ scores for the ABS-Arabic: *r*_ABS_ = 0.78, *p* < .001, *N* = 66.

## Discussion

The long-term outcomes of the FC-EIBI program that began before 5 years of age in children with ASD demonstrated continued positive impacts on development. In our three-year follow-up, the participants maintained the gains achieved from the program in many areas, including adaptive behavior. This provides further evidence that the participants did not exhibit developmental regression. However, some participants’ social skills declined, as measured on the ABS-Arabic. The regression of ABS-Arabic scores was specifically across social interaction, communication, and language subdomains. These findings suggest the possibility that the developmental regression of social ability gains obtained by the end of the FC-EIBI program could not be generalized to new areas of functioning. In particular, these results show that sheer developmental maturation is not the only driving factor in children’s improvements over time. The deprivation of social interaction opportunities as a result of the war may explain this regression.

The results of the present study are consistent with the results of McEachin et al.^60^ and Smith et al.^51^, in that these studies demonstrated maintenance of treatment several years after discontinuation of EIBI. In our study, parents were involved in the FC-EIBI program and hence the children may have maintained their gains because some forms of home-based ABA provision continued for those children after the FC-EIBI had formally ended.

Our finding is consistent with the results of Perry et al.^61^ in that ASD symptoms decreased significantly between intake and follow-up, and the effect size was large. Similarly, Smith et al.^51^ reported that the children continued to make progress after EIBI had ended. Some researchers (e.g., Steinhausen et al.^26^) have found that the long-term outcomes of some individuals with ASD are poor. However, our findings that strongly support the effectiveness of EIBI are similar to those obtained by Ben-Itzchak and Zachor^2^, in which the Autism Diagnostic Observation Schedule was used to examine autism symptom severity as a predictor of outcomes. The results showed substantial progress in all six developmental and behavioral domains after 1 year of intervention, and short-term behavioral treatment might enhance the adaptive functioning of children with ASD. This underscores the importance of EIBI in children with ASD. Our findings were also similar to those obtained by Estes et al*.*^5^, who found that children in the Early Start Denver Model maintained the gains that they had made in early intervention. However, in contrast to our findings, the participants in their study did not exhibit developmental regression. In addition, our findings are also dissimilar to the TRICARE report of their health care program for almost 9.5 million beneficiaries worldwide who showed no improvement in ASD symptoms over 12 months of enrollment in an ABA program and demonstrated worsening symptoms among 9% of participants^64^.

Though sample attrition may be unavoidable in a study of this kind^59^, we were lucky that we lost just one participant from the original cohort unlike Smith et al.^51^ where more than half of the original study participants were lost.

Our findings indicate that the place of residence in conflict zones may be considered a driving factor in children’s improvements over time. The children who were not displaced showed a higher level of improvement across the ABS-Arabic. Similarly, Tspilova et al.^57^ had outcomes when they compared long-term outcomes with children diagnosed with autism. They found that scores were associated with a geographic location in children when a country location was considered. While the participants were not located in a country affected by civil war, they nevertheless affirmed that location and outcomes might matter. The results of the current study strongly confirm how the Syrian civil war continues to have a large effect on children living in Syria. Countless Syrian children are affected by violence, displacement, loss of or separation from family members, and lack of access to vital services. For example, the United Nations estimates that 11 million people in Syria, including 4.7 million children and 1.3 million people with disabilities, require humanitarian assistance^39^.

Despite these results, our findings are encouraging, given that the participants are living in a region engaged in civil war. The participants received EIBI services through the Future Center, and 3 years after leaving the FC-EIBI program, they maintained their skills in several areas, despite the lack of EIBI services and living in an unstable region. The findings by Ben-Itzchak and Zachor^2^ involved children in stable conditions, whereas the community conditions of our participants are considered undesirable, yet their skills are maintained overall. Behavior analysts should be cautious when considering ideal conditions for services. Given the longitudinal outcomes of our study, it seems that there might be a greater need to consider how extenuating those conditions might be and still provide effective services to children with ASD.

Finally, the current study found a significant negative association between participants’ performance on the CARS, ABC, and ABS-Arabic and the number of weekly trials. These results emphasize the importance of continuing to support children in generalizing acquired skills in different settings. Although numerous studies have examined the effectiveness of EIBI in young children with ASD, few have focused on the family aspects of the program. In particular, war conditions and parent involvement in training^40–42^. More research from low- and middle-income countries and conflict zones is certainly needed to understand the feasibility, acceptability, and efficacy of behavioral interventions in non-Western societies. In light of these findings, more qualitative studies are required to explain these relationships.

The outcomes of the evaluation are generally positive; however, this study has several limitations. First, a limitation of the current study was the lack of a control group. Consequently, it cannot be concluded that the change in children in the current study was the result of the intervention implemented rather than other factors^51^. Studies including control groups of participants receiving other interventions are needed to draw meaningful conclusions^58^. Second, the sample size was limited to 66 children in Syria. Third, it focused on patients receiving care at a specialty clinic with expertise in EIBI, and the findings might not be generalizable to a wider population of children with ASD receiving other models of care. Finally, the control of other variables may have influenced the outcomes. We found that some improvements, such as the use of more dynamic methods, better formatted printed material, and increased fidelity between the content’s implementation and the prescribed activities, were needed^43,44^. In the future, researchers should increase the number of participants and use a more rigorous design such as randomized controlled trials. This may be a daunting task since this study focused on children in Syria. However, studying the external validity of the EIBI is important; thus, additional longitudinal studies would benefit children with ASD and their EIBI programs.

The results of this program evaluation might be reasonably generalized to other groups of children in similar organizations in Syria. The results of this study may help to implement EIBI in government-funded kindergartens. Additionally, replicating the FC-EIBI model in other Syrian regions that may lack these resources is possible. In the future, the FC-EIBI should continue to increase the quality of services and engage with community partners to develop national standards to guide EIBI practices. The program should continue to improve data collection and reporting systems, which enable a better understanding of who accesses EIBI and the gaps in the current service delivery model.

In conclusion, researchers have provided evidence of the effectiveness of EIBI in children with ASD. Our study provided further evidence of the importance of providing intensive behavioral interventions to children, particularly in Syria. Overall, we found that children maintained skills under undesirable conditions, highlighting why professionals might be encouraged to provide systematic, intensive instruction to young children diagnosed with ASD and navigate barriers (e.g., civil war regions), knowing that their efforts will be rewarded with therapeutic outcomes.

## Methods

### Setting and participants

Families were contacted by personal phone calls to participate in this follow-up study which was completed in 2013. We included 66 of 67 participants with ASD who participated in the original cohort^45^. Only one participant was excluded because of death. These children were enrolled for 2 years in the FC-EIBI program between 2008 and 2010. The participants exited the program 3 years before the current study. Their ages ranged from 5 to 10 years and 7 months old. Thirty-seven percent (*n* = 25) of the participants were aged 6 to 8 years; 28% (*n* = 18), 8.1 to 10 years; and 34%, (*n* = 23) 10.1 years or older. The center’s psychologist confirmed the diagnosis and IQ scores of the study participants by using the Diagnostic and Statistical Manual of Mental Disorders (4th ed., DSM-IV^46^) and the Wechsler Intelligence Scale for Children-IV IQ test and which were used in the initial study^45^. The mean IQ of the participants was 61 (range, 39–83), and some of the participants were receiving support services (e.g., speech, language, or occupational therapy) after enrolling in the FC-EIBI program. The frequency of trials was classified as: no training/week (*n* = 21), less than five trials/week (*n* = 21), and more than five trials/week (*n* = 24). These trials were conducted by either parents or clinicians.

Forty-six percent of the participants were prescribed medication (e.g., risperidone, atomoxetine, and depakine). In this study, we did not attempt to control pharmaceutical treatment (Supplementary Table 1).

In addition, many families of the participants were displaced to other cities during the civil war (*n* = 37). Almost 29% of families had a low educational level, 29% had a low economic level, 24% were divorced, and 18% were abandoned (Supplementary Table 1).

### Procedures

#### FC-EIBI program

The Future Center is currently operating an EIBI program, coined FC-EIBI, aimed at children aged 1–10 years diagnosed with ASD in two Syrian cities. In this center, most children begin the program between 2 and 4 years of age^45^. “The FC-EIBI program was designed to teach parents to serve as active co-therapists for their children and help the latter to be successful at school, home, and in the community. The program comprised the following processes: (a) theoretical education, (b) workshops and practice, (c) documentation and assessment, (d) communication and feedback, and (e) implementation”^45^. Between 2008 and 2010, parents received 5 h of coaching per week in their interventions and written instructions on how to conduct trials two times a week during the 2-year intervention. While the participating children received 25 h of EIBI per week, arriving daily at the center at 8:30 a.m. and departing at 1:30 p.m. A total of 10 lessons were received per day, each lasting 30 min, during which the targeted skills were taught. The mean number of skills in which children became proficient (i.e., goal reached) per child was 52/year, and the mean number of trials was 39 trials/week (range, 36–45) per child^45^. In the program, the most targeted skills were social interaction, communication and language, self-care, gross and fine motor skills, and cognitive and pre-academic skills, all of which were taught daily. Trials to identify children’s proficiency in each skill varied depending on their skill acquisition rates (*M* = 1 week).

### Design

We followed up on the initial study^45^ and evaluated the effects of the FC-EIBI program on the 66 participants using a longitudinal quasi-experimental design, assessing changes in participants’ scores for the ABC, CARS, and ABS-Arabic from 2010 to 2013. The assessors were all independent of the study.

### Instruments

The ABC, CARS, and ABS-Arabic scales were selected for this study because they were used in the initial study^45^, available in Arabic, and were the most appropriate and valid measures available. In the initial study, we computed a high level of internal consistency across the scales (α_ABC_ = 0.95; α_CARS_ = 0.98; α_ABS_ = 0.87). Additionally, there was high test–retest reliability across the scales (*r*_ABC_ = 0.96, *r*_CARS_ = 0.91, *r*_ABS_ = 0.87, *p* < .001).

We used the Arabic version of the ABC developed by Ghazal^47^. The ABC was completed independently by parents or teachers with 3 to 6 weeks of familiarity with a child. It took 10 to 20 min to complete and comprised 5 subscales and 57 items: releasing (12 items), sensory (9 items), language (12 items), body and object use (13 items), and social and self-help skills (11 items). Items were scored from 1 to 4, and the total score was obtained by adding the weights of the different areas. In prior research, scores greater than 67 points indicated ASD^37^; we used this score as the threshold value for ASD diagnosis. Higher scores indicated more autistic behavior symptoms.

The CARS assessed behavior across 14 domains severely affected by problems related to ASD and included a general category of impressions of ASD. The 15-item scale included: visual response, relating to people, emotional response, body use, object use, adaptation to change, activity level, listening response, level and consistency of intellectual relations, perceptive response, fear or anxiety, verbal communication, nonverbal communication, imitative behavior, and general impressions. The examiners scored 1 to 4 for each item (1 = age-appropriate behavior; 4 = severe deviance from age-appropriate behavior). The scores were summed to obtain a final score. The scores ranges were interpreted as: below 30 indicating that the child does not have ASD; 30 to 37 indicating mild to moderate ASD; and above 37 indicating severe ASD (CARS^52^;. In this study, a score of 30 or higher was the threshold for diagnosing ASD.

The ABS-Arabic was designed for the Arabic culture^53^. The ABS-Arabic was completed independently by a teacher familiar with the child for at least 6 weeks. The assessment was comprised of 6 subscales and 96 items: communication and language (16 items), social interaction (16 items), self-care (16 items), gross and fine motor skills (16 items), personal and emotional adaptation (16 items), and cognitive skills (16 items). Each domain has two subdomains, each of which has eight items. Items were scored using a scale ranging from 1 (age-appropriate behavior) to 4 (severe deviance from age-appropriate behavior). All item scores were summed up to provide a total score. Higher scores indicated lower adaptive behavior problems^53^.

### Fidelity assessment

The first author collected fidelity assessment data on the psychologist’s assessment procedures during 43% of the assessment sessions using a checklist that included the following steps: (a) providing a positive statement regarding performance in the previous video footage; (b) stating areas for improvement; (c) providing immediate feedback for both correct responses and errors; (f) asking if the psychologist has any questions; and (h) ending the session with a positive statement. The face validity of the checklist was assessed by asking two expert peers to review it. Fidelity assessment data were calculated as the percentage of steps performed correctly and were scored as 100%.

### Data analysis

The Statistics Package for Social Science v 29.0 was used for all statistical analyses. Descriptive statistics were expressed as means and ± standard deviations. To examine the children’s long-term course of development after the intervention, repeated measures ANOVAs were conducted^48^ with time points as a within-subjects factor including the pre-test scores as a covariate in the repeated measures ANOVA test. The results, along with effect sizes calculated using *η*^2^^49,50^. In addition, independent samples and the paired *t*-test were used to evaluate the social validity aspect and compare mean differences between the two measures. We also employed Spearman's Rank correlation coefficient to assess the relationship between the two variables.

### Ethical approval

This work was carried out following the Code of Ethics of the World Medical Association (Declaration of Helsinki) for experiments involving humans and its later amendments or comparable ethical standards. According to national and international regulations, the protocol was approved by the Ethics Committee from Damascus University, Department of psychology. Due to the affiliation change for the first author from Damascus University to Stockholm University, a new ethics review application (approval number 2022-01,262-01) was approved by the Swedish Ethical Review Authority.

The integrity of the participants was assured. The families were asked for permission to collect the relevant information for the study. Informed consent from all parents and/or legal guardians for participating children was obtained. Parents could interrupt their children’s participation at will. Furthermore, parents had access to the data and were able to select what data could be used or not, without penalties. The written consent form was presented and explained to clarify any doubts. Data protection has been considered.

## Supplementary Information

Supplementary Information.

## Data Availability

The datasets generated and analyzed during the current study are not publicly available due to some ethical reasons but are available from the corresponding author upon reasonable request.
