# Supplementary material for: A follow-up study of early intensive behavioral intervention program for children with Autism in Syria
Source: Sci Rep. 2023 Jan 2;13:70. doi: 10.1038/s41598-022-27198-4 (PMC9807639; doi:10.1038/s41598-022-27198-4)
Supplement: Supplementary file 1 — Supplementary Information. [file 41598_2022_27198_MOESM1_ESM.docx]

**Supplementary Tables**

| **Supplementary Table 1.**  Participants’ demographic characteristics | | | | | | | |
| --- | --- | --- | --- | --- | --- | --- | --- |
|  | | N | % |  | | N | % |
| Sex | Male | 38 | 58.2 | Prescribed  Medications | With | 31 | 46.3 |
|  | Female | 28 | 41.8 |  | Without | 35 | 53.7 |
| Age | 1–3 years | 25 | 37.3 | Family Civil Status | Married | 38 | 58.2 |
|  | 3–5 years | 18 | 28.4 |  | Divorced | 16 | 23.9 |
|  | 5 and more | 23 | 34.3 |  | Abandoned | 12 | 17.9 |
| Diagnosis | PDD-Nos | 9 | 13.4 | Family’s Economic Level | Poor | 19 | 28.4 |
|  | High function Autism | 8 | 11.9 |  | Good | 17 | 25.4 |
|  | Au + mild ID | 25 | 38.8 |  | Very good | 17 | 26.9 |
|  | Au + middle ID | 21 | 31.3 |  | Excellent | 13 | 19.4 |
|  | Au + severe ID | 3 | 4.5 |  |  |  |  |
| Sibling Participation | No participation | 18 | 28.4 | Acceptance Stages | Shock | 7 | 10.4 |
|  | Very little | 14 | 20.9 |  | Denial | 13 | 19.4 |
|  | Average | 18 | 26.9 |  | Seek Services | 18 | 28.4 |
|  | Large | 9 | 13.4 |  | Acceptance | 16 | 23.9 |
|  | A lot | 7 | 10.4 |  | Adjustment | 12 | 17.9 |
| Father’s Educational Level | Primary | 20 | 29.9 | Mother’s Educational Level | Primary | 20 | 29.9 |
|  | Middle | 16 | 23.9 |  | Middle | 17 | 26.9 |
|  | Secondary | 13 | 19.4 |  | Secondary | 15 | 22.4 |
|  | College | 17 | 26.9 |  | College | 14 | 20.9 |
| Number of Weekly Trials | No Training | 21 | 31.8 | Displacement | Home | 29 | 43.9 |
|  | <5 | 21 | 31.8 |  | Displaced | 37 | 56.1 |
|  | >5 | 24 | 36.4 |  |  |  |  |
| Total |  | 67 | 100% | Total |  | 67 | 100% |

^a^ Poor = < $150; Good = $150–250; Very good = $250–400; Excellent = > $400 per month in US dollars. ^b^ PDD-NOS: pervasive developmental disorder not otherwise specified; ID: intellectual disability; AU: autism

| **Supplementary Table 2.**  Pairwise Comparisons Pre-. Post-. and Follow-up Tests Among CARS. ABC and ABS-Arabic. | | | | | | |
| --- | --- | --- | --- | --- | --- | --- |
| (I) CARS | (J) CARS | Mean Difference (I-J) | Std. Error | *P* | 95% Confidence Interval | |
|  |  |  |  |  | Lower Bound | Upper Bound |
| 1 | 2 | 3.42^*^ | .17 | .000 | 3.02 | 3.83 |
|  | 3 | 5.32^*^ | .20 | .000 | 4.82 | 5.82 |
| 2 | 1 | -3.44^*^ | .17 | .000 | -3.83 | -3.02 |
|  | 3 | 1.89^*^ | .17 | .000 | 1.47 | 2.32 |
| 3 | 1 | -5.32^*^ | .20 | .000 | -5.82 | -4.82 |
|  | 2 | -1.89^*^ | .17 | .000 | -2.32 | -1.47 |
| (I) ABC | (J) ABC |  |  |  |  |  |
| 1 | 2 | 22.88* | 1.21 | .000 | 19.89 | 25.86 |
|  | 3 | 34.44* | 1.49 | .000 | 30.76 | 38.12 |
| 2 | 1 | -22.88* | 1.21 | .000 | -25.86 | -19.89 |
|  | 3 | 11.56* | .96 | .000 | 9.21 | 13.91 |
| 3 | 1 | -34.44* | 1.49 | .000 | -38.12 | -30.76 |
|  | 2 | -11.56* | .95 | .000 | -13.91 | -9.21 |
| (I) ABS-Arabic | (J) ABS-Arabic |  |  |  |  |  |
| 1 | 2 | -150.05* | 6.18 | .000 | -165.24 | -134.85 |
|  | 3 | -186.02* | 4.38 | .000 | -196.78 | -175.25 |
| 2 | 1 | 150.04* | 6.18 | .000 | 134.85 | 165.24 |
|  | 3 | -35.97* | 4.46 | .000 | -46.93 | -25.01 |
| 3 | 1 | 186.02* | 4.38 | .000 | 175.25 | 196.78 |
|  | 2 | 35.97* | 4.46 | .000 | 25.01 | 46.93 |
| * ^a^ ABC: autism behavior checklist; CARS: childhood autism rating scale; ABS-Arabic: adaptive behavioral scale-Arabic. ^b^N: total number of individuals in the sample; P: probability that measures the evidence against the null hypothesis. The mean difference is significant at the.05 level. ^**^ Adjustment for multiple comparisons: Bonferroni. | | | | | | |

| **Supplementary Table 3.** | | | | | | | | | | | |  |
| --- | --- | --- | --- | --- | --- | --- | --- | --- | --- | --- | --- | --- |
| T-test Comparisons Follow-up Tests Among CARS. ABC and ABS-Arabic | | | | | | | | | | | |  |
|  | Dicplaced | N | Mean | Std. Deviation | Mean Difference | *t* | *df* | *P* | | 95% Confidence Interval | | |
|  |  |  |  |  |  |  |  |  |  | Lower | Upper | |
| CARS.2013 | displaced | 29 | 33.76 | 5.57 | 5.05 | 4.02 | 64 | .000 | 2.55 | | 7.56 |  |
|  | Orginal home | 37 | 28.70 | 4.62 |  |  |  |  |  | |  |  |
| ABC.2013 | displaced | 29 | 99.52 | 25.98 | 27.24 | 4.60 | 64 | .000 | 15.41 | | 39.08 |  |
|  | Orginal home | 37 | 72.27 | 22.11 |  |  |  |  |  | |  |  |
| ABS-Arabic 2013 | displaced | 29 | 288.19 | 43.92 | 62.43 | 5.54 | 64 | .000 | 84.92 | | 39.94 |  |
|  | Orginal home | 37 | 225.76 | 47.22 |  |  |  |  |  | |  |  |

^a^ ABC: autism behavior checklist; CARS: childhood autism rating scale; ABS-Arabic: adaptive behavioral scale-Arabic. ^b^N: total number of individuals in the sample; *t:* t-test value; *df*: degree of freedom *P:* probability that measures the evidence against the null hypothesis.

| **Supplementary Table 4.**  Spearman's Rank correlation coefficient by the number of weekly trials | | | | | |
| --- | --- | --- | --- | --- | --- |
|  | | Weekly time | CARS 2013 | ABC 2013 | ABS-Arabic 2013 |
| Spearman's rho Weekly time | *r* | 1.00 | -.781^**^ | -.812^**^ | .780^**^ |
|  | Sig. (2-tailed) | . | .000 | .000 | .000 |
|  | N | 66 | 66 | 66 | 66 |
| ^a^**. Correlation is significant at the 0.01 level (two-tailed). *N*: total number of individuals in the sample; *r*: Pearson correlation coefficient. | | | | | |
